# Selection pressure by specialist and generalist insect herbivores leads to optimal constitutive plant defense. A mathematical model

**DOI:** 10.1002/ece3.10763

**Published:** 2023-12-04

**Authors:** Suman Chakraborty, Jonathan Gershenzon, Stefan Schuster

**Affiliations:** ^1^ Department of Bioinformatics, Matthias Schleiden Institute Friedrich Schiller University Jena Jena Germany; ^2^ International Max Planck Research School “Chemical Communication in Ecological Systems” Jena Germany; ^3^ Department of Biochemistry Max Planck Institute for Chemical Ecology Jena Germany

**Keywords:** generalist insects, glucosinolates, mathematical model, natural enemies, optimal constitutive defense, specialist insects

## Abstract

Brassicaceae plants have the glucosinolate–myrosinase defense system, jointly active against herbivory. However, constitutive glucosinolate (GLS) defense is observed to occur at levels that do not deter all insects from feeding. That prompts the question of why Brassicaceae plants have not evolved a higher constitutive defense. The answer may lie in the contrasting relationship between plant defense and host plant preference of specialist and generalist herbivores. GLS content increases a plant's susceptibility to specialist insects. In contrast, generalists are deterred by the plant GLSs. Although GLSs can attract the natural enemies (predators and parasitoids) of these herbivores, enemies can reduce herbivore pressure to some extent only. So, plants can be overrun by specialists if GLS content is too high, whereas generalists can invade the plants if it is too low. Therefore, an optimal constitutive plant defense can minimize the overall herbivore pressure. To explain the optimal defense theoretically, we model the contrasting host selection behavior of insect herbivores and the emergence of their natural enemies by non‐autonomous ordinary differential equations, where the independent variable is the plant GLS concentration. From the model, we quantify the optimal amount of GLSs, which minimizes total herbivore (specialists and generalists) pressure. That quite successfully explains the evolution of constitutive defense in plants from the perspective of optimality theory.

## Graphical abstract

Specialist herbivores are attracted by constitutive plant defense while generalists are deterred by such a defense. Therefore, there is an optimal level of plant defense chemicals. By a mathematical model, we determine that optimum.

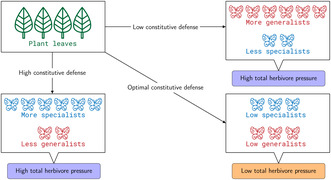

## 1 INTRODUCTION

Plants of the Brassicaceae family have a two‐component glucosinolate (GLS)‐myrosinase defense system to resist herbivory (Halkier & Gershenzon, 2006; Lazzeri et al., 2004; Wittstock et al., 2003). Although the glucosinolates (GLSs) themselves are not toxic, herbivory instigates GLS hydrolysis by myrosinase to produce toxic isothiocyanate products (Sun et al., 2019; Wittstock & Burow, 2010). The feeding insects adapted resistance (counter‐defense) techniques to avoid plant toxins (Jeschke et al., 2016; Schramm et al., 2012; Zou et al., 2016).

Specialists are usually less (or marginally) affected by plant defense (Li et al., 2000; Rohr et al., 2011; Sarosh et al., 2010), because they can circumvent the formation of isothiocyanates quite efficiently. Some GLS metabolizing specialists use preemptive detoxification of GLS, which provides an advantage over direct counter‐defense (Chakraborty et al., 2023; Jeschke et al., 2017). For example, *Pieris rapae* redirects GLS hydrolysis to form less toxic nitriles (Wittstock et al., 2004) and *Plutella xylostella* desulfates GLSs before hydrolysis (Ratzka et al., 2002). Sequestering specialists (Petschenka & Agrawal, 2016), such as turnip sawfly (*Athalia rosae* L.) and horseradish flea beetles (*Phyllotreta armoraciae*) rapidly absorb GLSs before hydrolysis (Müller et al., 2001; Sporer et al., 2021). In contrast, due to their inefficient counter‐defense techniques, generalists cannot avoid the exposure to isothiocyanates (Jeschke et al., 2016, 2017; Schramm et al., 2012). That is why plant defense is detrimental to generalists (Jeschke et al., 2021; Zalucki et al., 2021).

**FIGURE 1:**
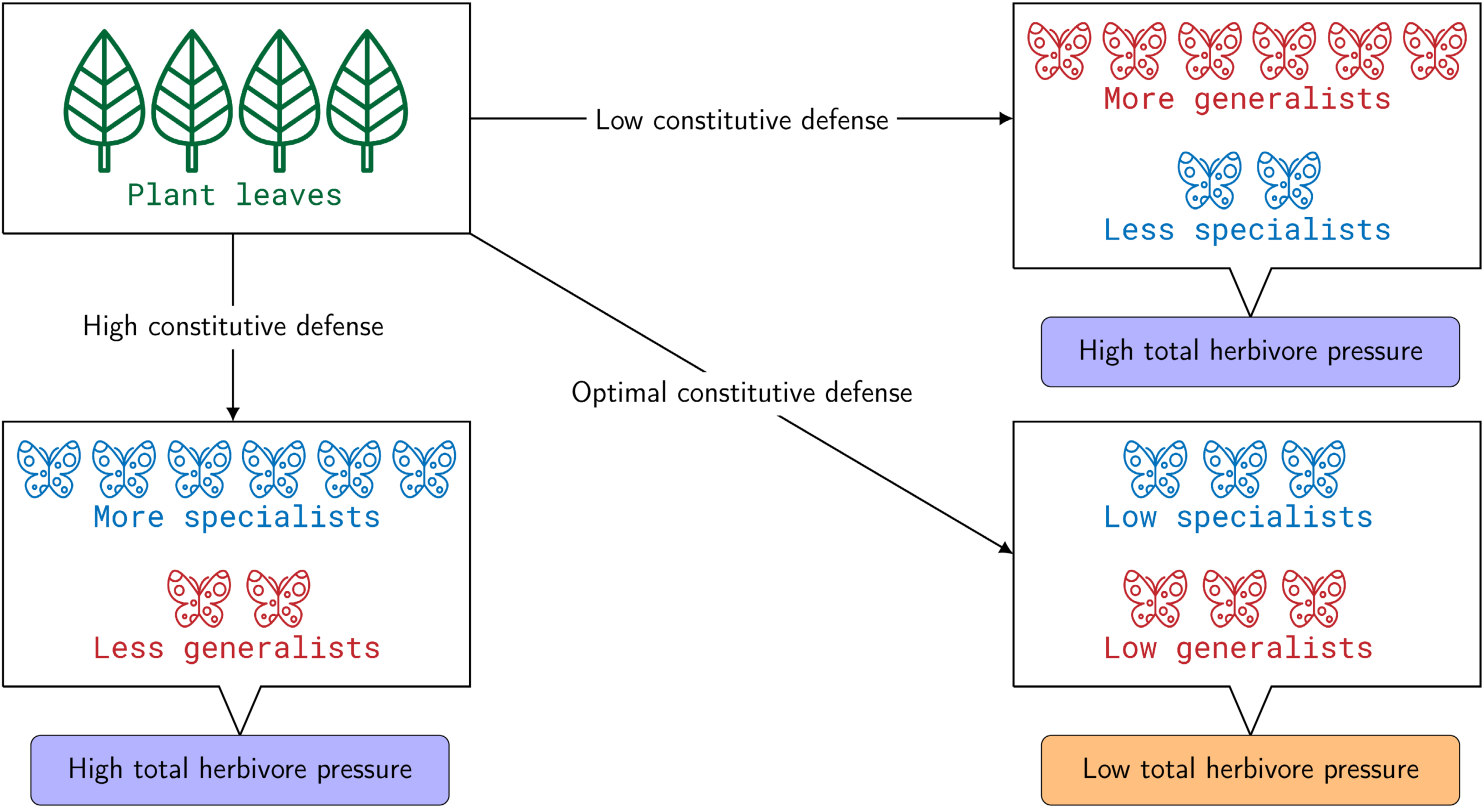
Contrasting host preference of insect herbivores vs. constitutive plant defense.

Intuitively, if plants store high amounts of defense substances (GLSs), the toxic effect (caused by isothiocyanates) could also become high. However, the constitutive GLSs are detectable throughout the Brassicaceae plants at a moderate level only (Textor & Gershenzon, 2009), where constitutive defense refers to the stored plant defense before the occurrence of herbivory (Dicke, 1998). That only moderate levels are stored in the unperturbed state becomes clear from the observation that upon herbivory, GLSs are usually induced (Karban & Myers, 1989; Textor & Gershenzon, 2009). So, the question is why plants did not evolve a higher level of constitutive defense? The contrasting relationship between plant defense and host plant preference by different group of insects can give the solution to this question (van der Meijden, 1996), as illustrated by the schematic diagram shown in Figure 1.

Specialist insects can cope with the toxin(s) of their preferred plants because they possess resistance (counter‐defense) mechanisms against the defense chemicals. They even use the GLS content (sometimes isothiocyanates, too) as a cue to identify plants for oviposition and feeding (Bidart‐Bouzat & Kliebenstein, 2008; Mewis et al., 2002; Miles et al., 2005; Renwick, 2002). For example, alkenyl glucosinolates stimulate feeding and oviposition by different types of specialist insects such as *Brevicoryne brassicae* (cabbage aphid), *Ceutorhynchus obstrictus* (cabbage seed weevil), *Dasineura brassicae* (brassica pod midges), *Delia radicum* (cabbage root flies), *Lipaphis erysimi* (turnip aphids), *Pieris rapae* (small white), and *Plutella xylostella* (diamondback moth) (Bidart‐Bouzat & Kliebenstein, 2008; Raybould & Moyes, 2001), GLSs stimulate the feeding and oviposition by *P. rapae* (Blau et al., 1978; Slansky Jr & Feeny, 1977) and are used for host recognition by *P. xylostella* (Badenes‐Perez et al., 2020). Even isothiocyanates act as an oviposition stimulant to *P. xylostella* (Renwick et al., 2006). Moreover, in wild‐type plants, larvae of *P. xylostella* are more abundant in lines with higher GLSs concentration (Kos et al., 2011; van der Meijden, 1996).

Generalists, on the contrary, are deterred by the GLS content of plants (Hopkins et al., 2009; Wittstock & Gershenzon, 2002). For example, GLS hydrolysis products of *Arabidopsis* plants are the major feeding deterrent to the generalists *Trichoplusia ni* and *Manduca sexta* (Barth & Jander, 2006), GLSs deter feeding by *Myzus persicae* (green peach aphid) on *Arabidopsis* (Kim & Jander, 2007). Although specialists and generalists have a contrasting host selection behavior, a host plant can be affected by both types of herbivores at the same time (Müller‐Schärer et al., 2004; Strauss & Irwin, 2004; van der Meijden, 1996). For example, GLSs of *Brassica nigra* deter generalist herbivores (such as snails and slugs), which leads to an increased load of *Brevicoryne brassicae*, a specialist aphid (Lankau, 2007). Therefore, the intensity of constitutive plant defense has the potential to control the total herbivore pressure on plants (Louda & Mole, 1991; Müller‐Schärer et al., 2004; Strauss & Irwin, 2004; van der Meijden, 1996).

Hydrolysis products of GLSs, notably isothiocyanates and also nitriles can recruit natural enemies (such as parasitoids and predators) on the Brassicaceae hosts (Blande et al., 2007; Mumm et al., 2008; Reddy et al., 2002). For example, nitriles attract the parasitoid wasp *Cotesia rubecula* in *Pieris rapae* infested *Arabidopsis* plants (Van Poecke et al., 2001) and *Trichogramma chilonis* wasps are recruited by isothiocyanates in *Plutella xylostella* infested GLS containing plants (Reddy et al., 2002). Herbivore pressure is obviously reduced by the emergence of natural enemies (Fergola & Wang, 2011; Liu et al., 2009). That is why attracting the natural enemies is considered as an indirect form of plant defense (Dicke & Baldwin, 2010).

Mathematical modeling is a useful tool to understand the kinetics of plant defense compounds (Hanschen et al., 2018; Hebert et al., 2022; Knoke et al., 2009) or any other toxic substrates (Schäuble et al., 2013; Schuster et al., 2019). Those defense compounds play pivotal roles in controlling herbivore populations (Fergola & Wang, 2011; Liu et al., 2009). Evolutionary roles of these defense compounds are explained by models, based on optimality principles (Hamilton et al., 2001; Siemens et al., 2010; Stamp, 2003; van der Meijden, 1996; Zhang & Jiang, 2006). For example, models explain the relation between plant defense and risk of herbivory (Åström & Lundberg, 1994), predict that the fast‐growing plants cannot have a high amount of defense (de Jong, 1995) and also suggest the optimal strategy for constitutive defense or induced defense or no defense against herbivory (Ito & Sakai, 2009).

Here, we propose a model based on non‐autonomous ordinary differential equations (ODEs), which describes the contrasting host selection behavior by specialist and generalist insect herbivores, as well as immigration of natural enemies with respect to the increasing constitutive plant defense (GLS) concentration (Louda & Mole, 1991; van der Meijden, 1996). In the model, we express total herbivore pressure as a function of plant GLS content. By that function, we prove that the total herbivore pressure is minimum at an optimal amount of GLSs. Thus, our results are indicative of an optimal trade‐off in the evolution of constitutive plant defense. The model explains why keeping the leaves less defended (constitutively) is practically beneficial for plants, which is a common natural phenomenon.

## 2 METHOD AND RESULTS

Let $S\left(D\right)$ be the attracted specialist population, $G\left(D\right)$ be the generalist population remaining on the plant (or patch of host plants) after deterrence and $N\left(D\right)$ be the population of immigrated natural enemies at the constitutive defense (GLS) level D. Let specialists are attracted at a rate α per unit plant defense, whereas generalists per capita are deterred at a rate β per unit plant defense. For simplicity, we assume that the natural enemies (affecting both specialists and generalists) are attracted with a constant factor, which we denote by γ, that is, natural enemies increase linearly with the plant defense. Since natural enemies reduce the herbivore pressure by predation or parasitism, let μ and η be the per capita mortality rate (or death rate) of specialist and generalist herbivores, respectively, caused by the natural enemies. The differential equations read:

(1a) $$\frac{dS}{dD}=\mathrm{\alpha D}-\mathrm{\mu SN}$$

(1b) $$\frac{dG}{dD}=-\mathrm{\beta GD}-\mathrm{\eta GN}$$

(1c) $$\frac{dN}{dD}=\gamma$$

The mortality (or death) of herbivores is represented by bilinear functions (μSN and ηGN) in Equations (1a, 1b) and (2a, 2b), which is frequently used in models of prey–predator interactions (Goel et al., 1971; Lotka, 1925; Volterra, 1931). The deterrence of generalists is also a bilinear function (βGD) in Equation (2a, 2b), because the number of deterred insects is proportional to the generalist population size and the concentration of plant defense. The initial conditions for *S* and *N* are assumed as $S\left(D=0\right)=0$ and $N\left(D=0\right)=0$, meaning that no attracted specialists nor natural enemies are present without any plant defense.

**FIGURE 2:**
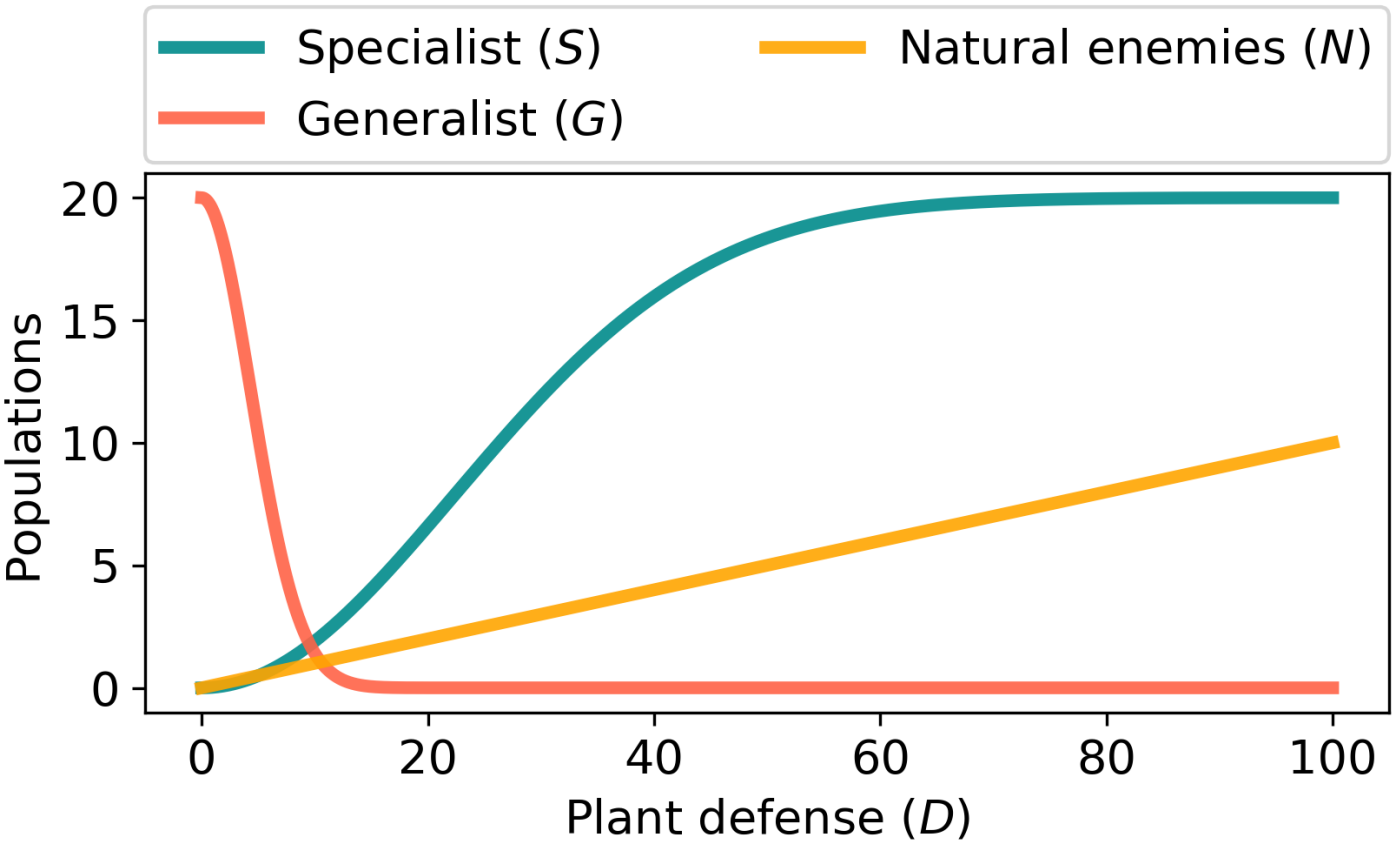
Populations of specialists, generalists, and natural enemies versus increasing plant defense (GLS). Parameters: α=0.04,β=0.05,γ=0.1,μ=0.02,η=0.03.

It is an interesting question whether specialists or generalists are more affected by natural enemies. Some specialists sequester toxins from plants to protect themselves against enemies (Petschenka & Agrawal, 2016; Sporer et al., 2021). At this stage, we do not use any order relation among μ and η. The dependent variables S,G and N are plotted versus the independent variable D in Figure 2 for some definite parameter values.

Model (1) can be simplified as follows, where the third ODE has been integrated:

(2a) $$\frac{dS}{dD}=\mathrm{\alpha D}\left(1-\frac{\theta }{\alpha }S\right),\;\text{where}\;\theta =\mu \gamma$$

(2b) $$\frac{dG}{dD}=-\left(\beta +\delta \right)\mathrm{GD},\;\text{where}\;\delta =\eta \gamma$$

(2c) N=γD

As specialists are attracted by constitutive plant defense, the derivative $\frac{dS}{dD}$ should be non‐negative (≥0). The fixed point for the specialist population (S) is $\frac{\alpha }{\theta }$, that is, $\frac{dS}{dD}=0$ at $S=\frac{\alpha }{\theta }$. Therefore, $\frac{\alpha }{\theta }$ can be considered as the carrying capacity of specialists, because it represents the maximum number of attracted specialists on a host plant (or patch of host plants).

Remark 1 If $\frac{\alpha }{\theta }$ is very low (→0), the specialist population does not grow. That occurs only when the predation (or parasitism) by natural enemies is very strong.

By solving Equation (2a, 2b), we can write S and G as functions of D:

(3a) $$S\left(D\right)=\frac{\alpha }{\theta }\;\left(1-e^{\frac{-\theta D^{2}}{2}}\right),\;\text{proof is given in Appendix}\;A.1$$

(3b) $$G\left(D\right)=G_{0}\;e^{-\frac{\left(\beta +\delta \right)D^{2}}{2}},\;\text{proof is given in Appendix}\;A.2$$

where $G_{0}=G\left(D=0\right)\geq 1$ is the maximum generalist population if the host plant (or patch of host plants) does not show any defense.

The total herbivore pressure on the host plant(s) is $S\left(D\right)+G\left(D\right)$, denoted by $T_{H}$. From Equations (3a, 3b), $T_{H}$ can be written as a function of D:

(4) $$T_{H}=\frac{\alpha }{\theta }\;\left(1-e^{\frac{-\theta D^{2}}{2}}\right)+G_{0}\;e^{-\frac{\left(\beta +\delta \right)D^{2}}{2}}$$

From Equation (4), it can be derived that $T_{H}$ is minimum at an optimal defense:

(5) $$D_{\mathrm{opt}}=\sqrt{\frac{2}{\beta +\delta -\theta }\ln\frac{\left(\beta +\delta \right)G_{0}}{\alpha }},\;\text{given}\;\beta +\delta >\theta \;\text{and}\;\left(\beta +\delta \right)G_{0}>\alpha$$

The proof is given in Appendix A.3. Since plant defense is more detrimental to generalists, the sum of the deterrence and mortality rates of generalist herbivores is higher than the mortality and attraction rate of specialist herbivores (Hopkins et al., 2009; Lankau, 2007; van der Meijden, 1996), that is, β+δ>θ and $\left(\beta +\delta \right)G_{0}>\alpha$, where $G_{0}\geq 1$.

**FIGURE 3:**
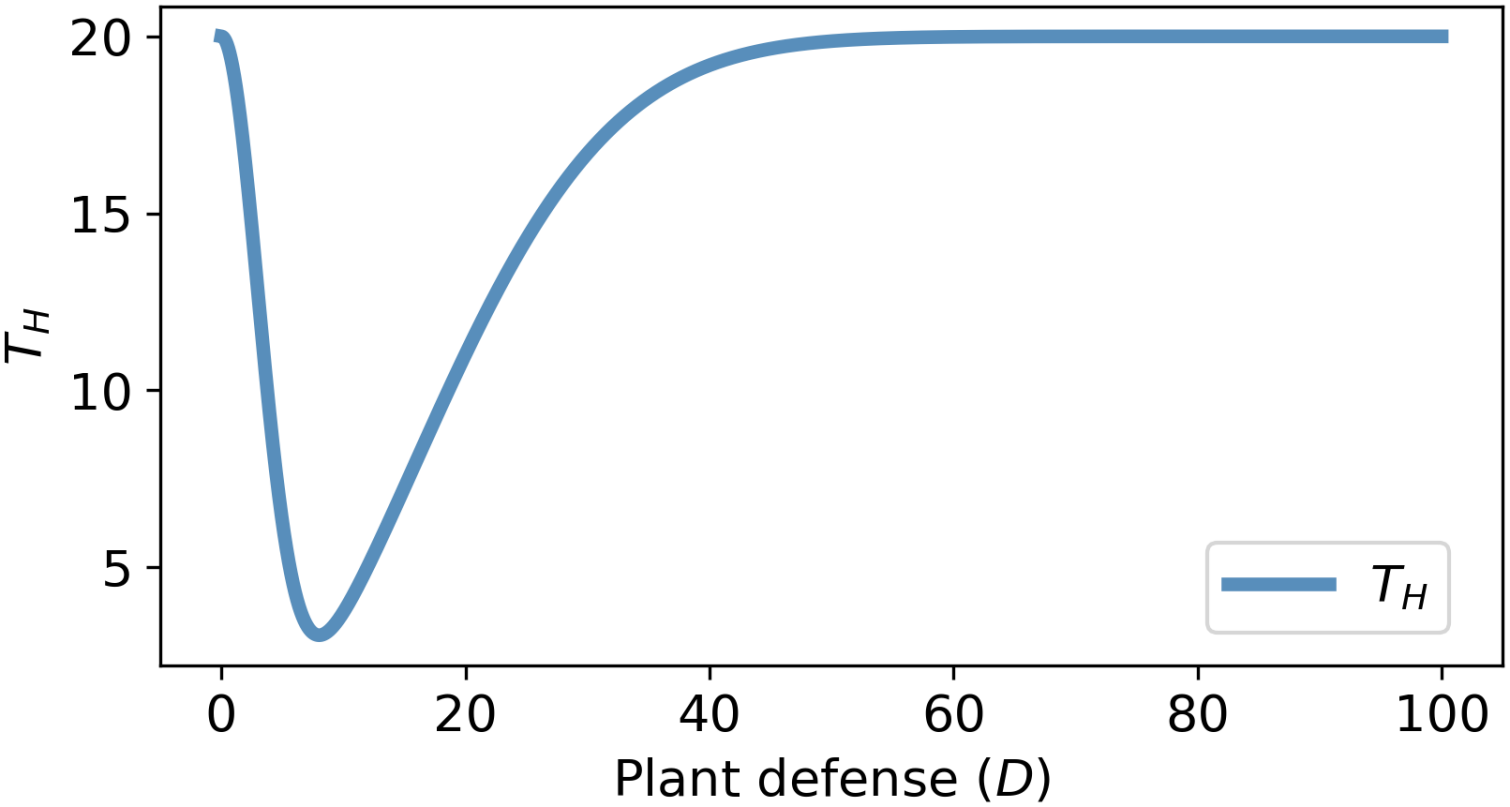
Total herbivore pressure ($T_{H}$) versus plant defense (GLS). θ=0.002,δ=0.003, $\frac{\alpha }{\theta }=G_{0}=20$. Other parameters are the same as in Figure 2.

Thus, Equation (5) proves that an optimal constitutive defense ($D_{\mathrm{opt}}$) allows the host plant (or patch of host plants) to minimize the total herbivore pressure, shown in Figure 3.

**FIGURE 4:**
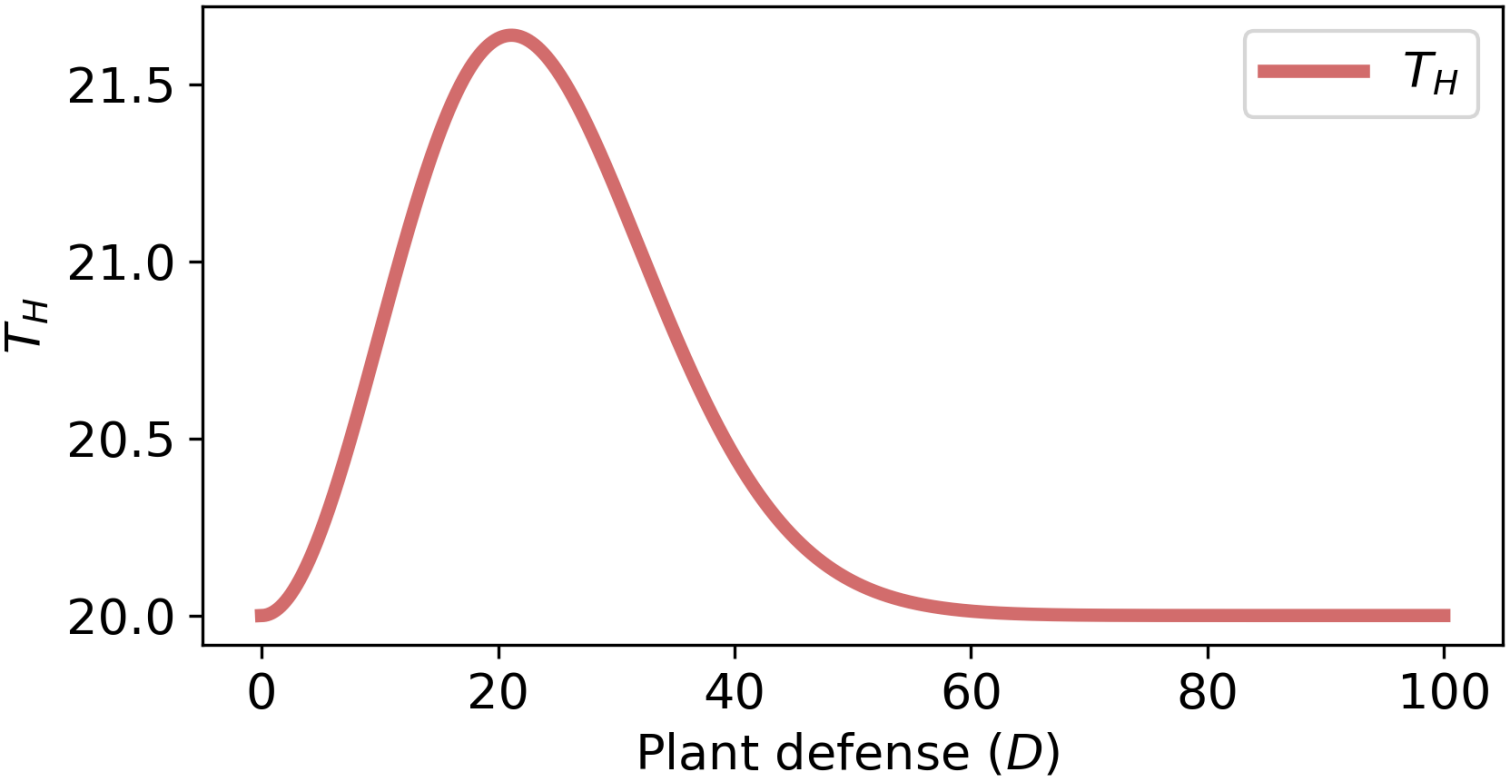
Total herbivore pressure ($T_{H}$) versus plant defense at θ>β+δ and $\alpha >\left(\beta +\delta \right)G_{0}$ (a what‐if situation). Parameters: α=0.05,θ=0.0025,β=0.001,δ=0.001 and $\frac{\alpha }{\theta }=G_{0}=20$.

Remark 2 In a hypothetical or what‐if situation, if θ>β+δ and $\left(\beta +\delta \right)G_{0}>\alpha$, then plant defense cannot reduce the total herbivore pressure below its initial value. On the contrary, the total herbivore pressure increases from its initial value to reach a certain maximum at some level of plant defense, shown in Figure 4. That would nullify the basic requirement of plant defense. However, experimental results suggest that most plants have some level of defenses, and the condition β+δ>θ or δ is practically always fulfilled (Hopkins et al., 2009; Lankau, 2007; van der Meijden, 1996).

**FIGURE 5:**
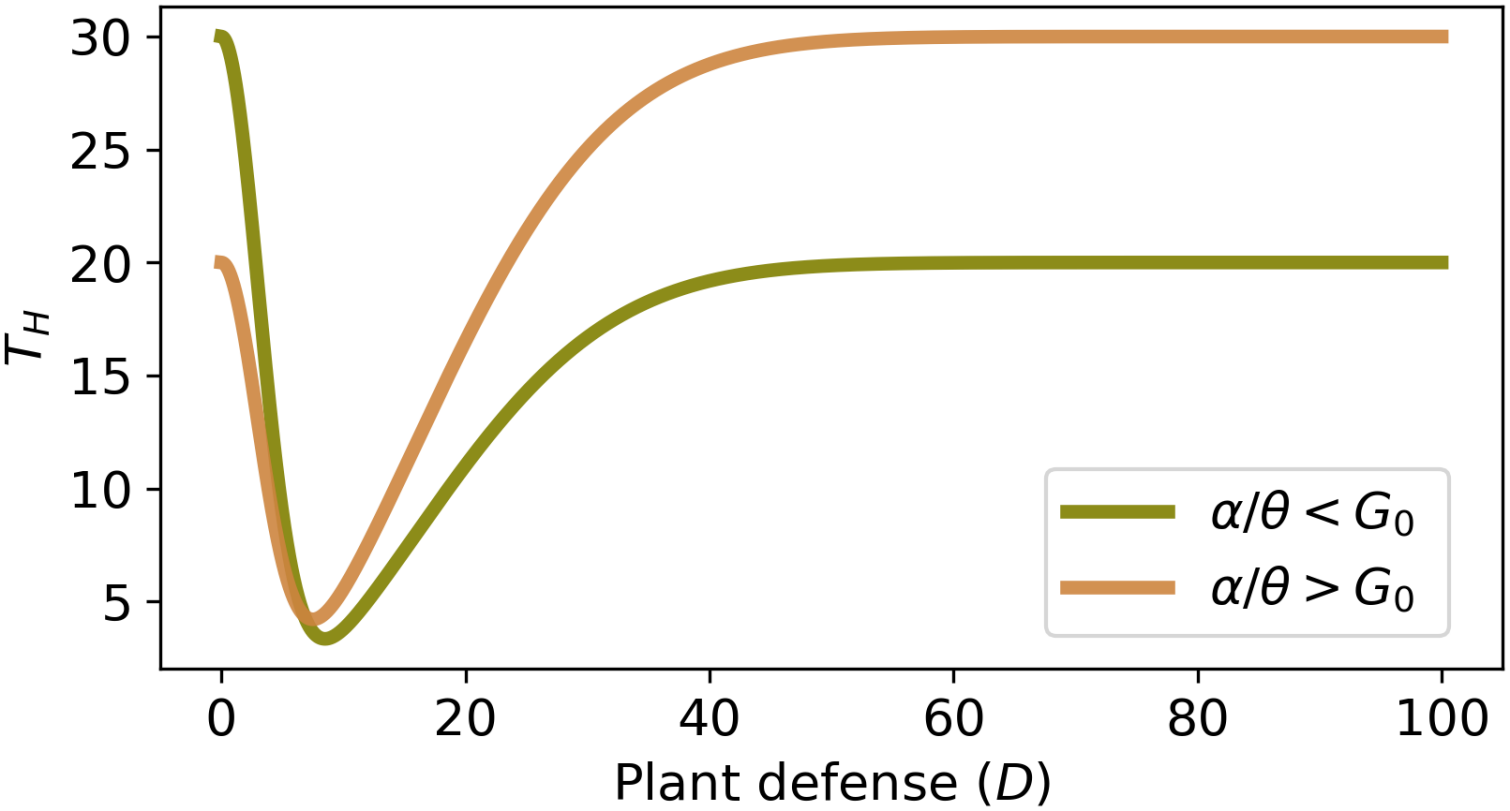
Total herbivore pressure ($T_{H}$) versus plant defense at $\frac{\alpha }{\theta }\neq G_{0}$. Parameters: θ=0.002,β=0.05 and δ=0.003.

Remark 3 Note that the most likely case is $\frac{\alpha }{\theta }\neq G_{0}$. Here, we assumed $\frac{\alpha }{\theta }=G_{0}$ in the Figures 2 and 3 in order that S and G reach the same maximum values. The outcome of our study (i.e., optimal constitutive defense, $D_{\mathrm{opt}}$ in Equation (5)) will not change in the case $\frac{\alpha }{\theta }\neq G_{0}$, as long as they are both non‐zero, see Figure 5.

**FIGURE 6:**
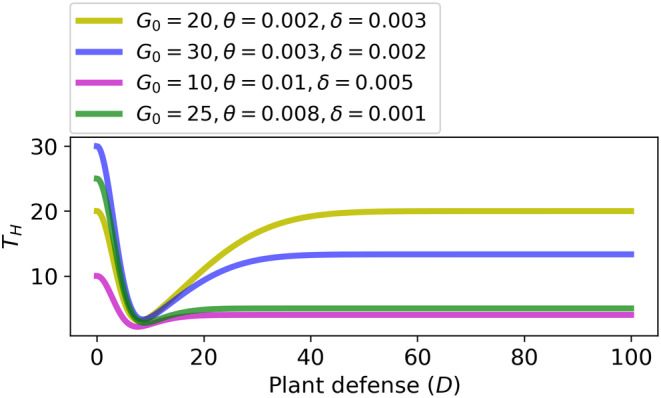
Robustness of the optimum with respect to the strength of predation or parasitism, that is, parameters θ and δ. Parameters α,β are the same as in Figure 2.

Remark 4 The predation (or parasitism) by natural enemies can be higher (or lower) on specialists than generalists. However, the optimum $D_{\mathrm{opt}}$ in Equation (5) is not affected by the strength of predation or parasitism, as illustrated in Figure 6.

## 3 DISCUSSION

We developed a general model (1) to explain evolution of constitutive defense, applicable not only to the Brassicaceae plant family, but to all plants with chemical defenses. In nature, constitutive defenses are present in most plants (Gershenzon & Ullah, 2022; Swain, 1977; Vickery, 2010). For example, caffeine in coffee, tea, cacao, and kola (Ashihara & Crozier, 1999; Kim & Sano, 2008), nicotine in tobacco (Steppuhn et al., 2004), terpenes and formylated phloroglucinol compounds (FPCs) in *Eucalyptus grandis* (Henery et al., 2008), morphine in opium poppy (Katherine et al., 2009), benzoxazinoids in the Gramineae family (Sicker et al., 2000), saponins in many dicotyledonous plants (Osbourn et al., 2003). Even plants edible by humans involve some defense chemicals, which can be tolerated due to their low amounts and are often sensed as flavors.

Our analysis and results help to conclude that constitutive defense levels are likely a product of natural selection to optimize defenses against two different kinds of herbivores. This comes about by a complex interplay between plants, specialist and generalist herbivores and natural enemies of the herbivores. A similar result was obtained earlier, using a graphical approach based on ad hoc dose–response curves (van der Meijden, 1996). Here, we have made this analysis more quantitative by using differential equations.

We are unaware of earlier deterministic ODE models contrasting host selection behavior of insects, controlled by plant defense. To initiate the modeling process by a fundamental model, we kept it simple and analytical. However, several advancements can be made for qualitative and numerical analysis. For example, the growth Equation (1c) for natural enemies is very simple (Figure 2) and can be improved. Demographic factors can be added to the Equations (1a), (1b) and (1c). Natural enemies can also be specialists or generalists, which affects their predation (or parasitism) behavior (Ghosh et al., 2022; Sheehan, 1986; Sun et al., 2019). Furthermore, foraging costs are not included in our model, which could be associated to the populations of insect herbivores as well as natural enemies (Parker & Smith, 1990; Schoener, 1971). So, incorporating these phenomena can considerably improve our model 1.

For a holistic understanding, it is worthwhile including metabolic costs of producing defense chemicals to observe the effect on plant fitness, that is, expanding our model (1) to include optimal defense theory (ODT) (Kessler & Halitschke, 2009; Martinez‐Swatson et al., 2019; McKey, 1974; Zangerl & Rutledge, 1996). Theoretically, the trade‐off between benefit and cost may lead plants to an optimal strategy of not investing in constitutive defense if damage by herbivory is not severe (Ito & Sakai, 2009). However, herbivory is usually severe for plants, so that most plants do produce defense chemicals (see above). In addition, we assumed the costs of producing defense chemicals to be negligible in comparison with specialist pressure.

Plants synthesize the constitutive defense compounds during their normal course of development and growth, notably in the absence of herbivory. These compounds are destined for a basic or initial protection to plants at the commencement of herbivory (Gatehouse, 2002; Wittstock & Gershenzon, 2002). Moreover, plant defense can be induced by herbivory (Agrawal, 1998; Karban, 2011; Karban & Myers, 1989; Textor & Gershenzon, 2009). For example, defense can be induced in cotton seedlings against herbivory by mites (Karban & Carey, 1984), feeding by tobacco horn worm (*Manduca sexta*) resulted in the induction of proteinase inhibitors in potato (*Solanum tuberosum*) or tomato (*Lycopersicon esculentum*) leaves (Schaller & Ryan, 1996), and GLSs are induced in *Arabidopsis lyrata* and *Brassica oleracea* plants in response to herbivory by small white (*Pieris rapae*) larvae (Agrawal & Kurashige, 2003). Interestingly, herbivory‐induced plant defense may depend on the types of feeding insect herbivores (Ali & Agrawal, 2012; Textor & Gershenzon, 2009). This effect is not incorporated yet into our model (1). However, the model can be extended in the future to envisage the optimal induction of plant defense, led by contrasting host preference by different insect groups. Then, not only the effect of the defense level (D) on the generalist pressure (G) but also the inverse effect will be considered.

Indirect defense is crucial for plants to kill specialist insect herbivores, because it recruits natural enemies. As discussed in Remark 1, a high predation (or parasitism) rate by natural enemies can arrest growth in a specialist population. An alternative method of reducing crop‐infesting insects is intercropping. An individual plant is less apparent (i.e., less susceptible to discovery) when growing next to the plants of other species (Feeny, 1976). Diversity of vegetation helps plants to escape or reduce herbivory (Feeny, 1976, 1977). For example, Brussels sprout plants in weed‐free soil are more susceptible to *B. brassicae* and other crucifer‐feeding insects than those grown among weeds (Smith, 1976). Intercropping particularly affects the specialist insects more (Root, 1973; Vandermeer, 1989) by altering plant odor (Finch & Collier, 2000) or masking the odor of host plants by associated plants (Tahvanainen & Root, 1972). Moreover, diversification of agroecosystems can increase the population of generalist enemies to kill insect herbivores (Sheehan, 1986). About 44% higher abundance of natural enemies and 54% higher herbivore mortality were reported in high‐diversity than low‐diversity agroecosystem (Letourneau et al., 2011).

Application of insecticides is another way to deter and kill crop‐infesting insects (Zhang et al., 2022). In a sense, those insecticides act as artificial enemies. One conclusion of the present study is that insecticides should be used against specialists (relevant for the cultivated crop), rather than against generalists. However, insecticide resistance comes up as a major problem for crop protection (Guedes et al., 2016). For example, the Colorado potato beetle (*Leptinotarsa decemlineata*) is resistant to 52 different compounds from all the major insecticide classes (Alyokhin et al., 2008) and several cases of insecticide resistance are noticed in *Pieris rapae* (Chou et al., 1984). Moreover, insecticides (especially, the synthetic ones) are detrimental for our environment, raising serious public health issues (Cassereau et al., 2017; Mansour et al., 2017). Therefore, a natural and sustainable way of farming crops is to let the population of natural enemies grow (Caltagirone, 1981), so that indirect plant defense can act smoothly.

## AUTHOR CONTRIBUTIONS

**Suman Chakraborty:** Conceptualization (lead); formal analysis (lead); investigation (lead); methodology (lead); software (lead); validation (equal); visualization (lead); writing – original draft (lead); writing – review and editing (equal). **Jonathan Gershenzon:** Project administration (supporting); supervision (supporting); validation (equal); writing – review and editing (equal). **Stefan Schuster:** Formal analysis (supporting); funding acquisition (lead); investigation (supporting); methodology (supporting); project administration (lead); supervision (lead); validation (equal); writing – review and editing (equal).

## CONFLICT OF INTEREST STATEMENT

The authors declare that the research was conducted in the absence of any commercial or financial relationships that could be construed as a potential conflict of interest.

## Supporting information


Data S1.

## Data Availability

The original contributions presented in the study are included in the article. The source code for producing the Figures is provided in the supplementary material. Further inquiries can be directed to the corresponding author.

## APPENDIX A

### A.1 Solution of Equation (2a)

$$\begin{array}{l} \;\frac{dS}{dD}=\mathrm{\alpha D}\left(1-\frac{\theta }{\alpha }S\right)\\ \;\Rightarrow \int \frac{dS}{1-\frac{\theta }{\alpha }S}=\int \mathrm{\alpha D}\;dD\\ \;\Rightarrow \int \frac{\alpha dP}{\mathrm{\theta P}}=-\int \mathrm{\alpha D}\;dD,\;\text{where}\;P=1-\frac{\theta }{\alpha }S\Rightarrow dS=-\frac{\alpha dP}{\theta }\\ \;\Rightarrow \frac{\alpha \ln P}{\theta }=-\frac{\alpha D^{2}}{2}+K,\;\text{where}\;K\;\text{is an arbitrary constant}\\ \Rightarrow \frac{\alpha \ln\left(1-\frac{\theta }{\alpha }S\right)}{\theta }=-\frac{\alpha D^{2}}{2},\;\text{where}\;K=0,\text{because}\;S\left(0\right)=0\\ \;\Rightarrow 1-\frac{\theta }{\alpha }S=e^{-\frac{\theta D^{2}}{2}}\\ \;\Rightarrow S=\frac{\alpha }{\theta }\left(1-e^{-\frac{\theta D^{2}}{2}}\right),\;i.e.\;\text{the solution},\text{Equation}\;\left(3a\right) \end{array}$$

### A.2 Solution of Equation (2b)

$$\begin{array}{l} \;\frac{dG}{dD}=-\left(\beta +\delta \right)\mathrm{GD}\\ \Rightarrow \int \frac{dG}{G}=-\int \left(\beta +\delta \right)D\;dD\\ \;\Rightarrow \ln G=-\frac{\left(\beta +\delta \right)D^{2}}{2}+L,\;\text{where}\;L\;\text{is an arbitrary constant}\\ \;\Rightarrow \ln G=-\frac{\left(\beta +\delta \right)D^{2}}{2}+\ln G_{0},\;\text{where}\;L=\ln\;G_{0},\text{because}\;G\left(0\right)=G_{0}\\ \;\Rightarrow G=G_{0}\;e^{-\frac{\left(\beta +\delta \right)D^{2}}{2}},\;i.e.\;\text{the solution},\text{Equation}\;\left(3b\right) \end{array}$$

### A.3 Proof of optimal constitutive defense, Equation (5)

$$\begin{array}{l} \;\frac{dT_{H}}{dD}=\alpha {\mathrm{De}}^{\frac{-\theta D^{2}}{2}}-\left(\beta +\delta \right)G_{0}{\mathrm{De}}^{\frac{-\left(\beta +\delta \right)D^{2}}{2}},\;\text{derivative of Equation}\;\left(4\right)\\ \;\frac{dT_{H}}{dD}=0,\;\text{to find the optimum}\;D\\ \;\Rightarrow \alpha {\mathrm{De}}^{\frac{-\theta D^{2}}{2}}=\left(\beta +\delta \right)G_{0}{\mathrm{De}}^{\frac{-\left(\beta +\delta \right)D^{2}}{2}}\\ \Rightarrow e^{\frac{\left(\beta +\delta -\theta \right)D^{2}}{2}}=\frac{G_{0}\left(\beta +\delta \right)}{\alpha },\;\text{where}\;D\neq 0\;\text{and}\;D↛\infty \\ \;\Rightarrow D^{2}=\frac{2}{\beta +\delta -\theta }\;\ln\frac{\left(\beta +\delta \right)G_{0}}{\alpha },\;\text{where}\;\beta +\delta >\theta \;\text{and}\;\left(\beta +\delta \right)G_{0}>\alpha \\ \;\Rightarrow D_{\mathrm{opt}}=\sqrt{\frac{2}{\beta +\delta -\theta }\;\ln\frac{\left(\beta +\delta \right)G_{0}}{\alpha }},\;\text{where}\;D_{\mathrm{opt}}\;\text{is the critical point} \end{array}$$

To prove that $D_{\mathrm{opt}}$ is the minimum, we check where $\frac{dT_{H}}{dD}>0$ and $\frac{dT_{H}}{dD}<0$.

$$\begin{array}{l} \;\frac{dT_{H}}{dD}>0\\ \Rightarrow \alpha {\mathrm{De}}^{\frac{-\theta D^{2}}{2}}>\left(\beta +\delta \right)G_{0}{\mathrm{De}}^{\frac{-\left(\beta +\delta \right)D^{2}}{2}}\\ \Rightarrow e^{\frac{\left(\beta +\delta -\theta \right)D^{2}}{2}}>\frac{G_{0}\left(\beta +\delta \right)}{\alpha },\;\text{where}\;D\neq 0\;\text{and}\;D↛\infty \\ \;\Rightarrow D^{2}>\frac{2}{\beta +\delta -\theta }\;\ln\frac{\left(\beta +\delta \right)G_{0}}{\alpha },\;\text{where}\;\beta +\delta >\theta \;\text{and}\;\left(\beta +\delta \right)G_{0}>\alpha \\ \;\Rightarrow D>\sqrt{\frac{2}{\beta +\delta -\theta }\;\ln\frac{\left(\beta +\delta \right)G_{0}}{\alpha }}=D_{\mathrm{opt}} \end{array}$$

Therefore, $T_{H}$ is strictly monotonic increasing in the interval $D_{\mathrm{opt}}<D<\infty$. Similarly, $\frac{dT_{H}}{dD}<0$ for $D<D_{\mathrm{opt}}$. That means $T_{H}$ is strictly monotonic decreasing in the interval $0<D<D_{\mathrm{opt}}$. Thus, it is proved that $T_{H}$ is minimum at $D_{\mathrm{opt}}$, as shown in Equation (5) and Figure 3.
