# Supplementary material for: Selection pressure by specialist and generalist insect herbivores leads to optimal constitutive plant defense. A mathematical model
Source: Ecol Evol. 2023 Dec 4;13(12):e10763. doi: 10.1002/ece3.10763 (PMC10695761; doi:10.1002/ece3.10763)
Supplement: Supplementary file 1 — Data S1. [file ECE3-13-e10763-s001.pdf]

Supplementary material

Latex and python codes of the Figures in our manuscript "Selection pressure by specialist and generalist insect herbivores leads to optimal constitutive plant defense. A mathematical model".

1 Latex code of Figure 1

```
% Import the "tikz" package in the Latex file
\usetikzlibrary{arrows.meta}
\tikzset{%
  >={Latex[width=2mm,length=2mm]},
  % Specifications for style of nodes:
            base/.style = {rectangle, rounded corners, draw=black,
                           minimum width=5cm, minimum height=1cm,
                           text centered, font=\sffamily},
  % Color and width of different rectangular boxes
  activityStarts1/.style = {base, minimum width=5cm, fill=magenta!30},
  activityStarts2/.style = {base, minimum width=5cm, fill=cyan!40},
       startstop/.style  = {base, minimum width=5cm, fill=red!30},
    activityRuns/.style  = {base, minimum width=5cm, fill=green!30},
         process1/.style = {base, minimum width=6cm, fill=orange!50},
         process2/.style = {base, minimum width=6cm, fill=blue!30},
         process3/.style = {base, minimum width=5cm, fill= orange!50},
         suman/.style    = {base, minimum width=5cm, fill=white!30},
         papai/.style    = {base, minimum width=5cm, fill=Gray!30}
}
%%%%%%%%%%%%%%%%%%%%%%%%%%%%%%%%%%%%%%%%%%%%%%%%%%%%%%%%%%%%%%%%%%%%%%%%%%%%%%%%%%%%%%%%%%%%%%%%%

% Begin code with \begin{tikzpicture}
[node distance=1.5cm,
every node/.style={fill=white, font=\sffamily}, align=center]
\node[inner sep=0pt] (L) at (12,9) []{\includegraphics[width=0.4\columnwidth]{Leaves.png}};
\draw[-, thick] (8.6,8.2) -- (8.6,11.5) -- (15.3,11.5)--(15.3,8.2) -- (8.6,8.2);

\node[inner sep=0pt] (G) at (26.5,9) []{\includegraphics[width=0.4\columnwidth]{generalists.png}};
% generalists.png is a image file, created seperately.

% Draw the arrows
\draw[->, thick] (15.3, 9.85) -- (23.1, 9.85)

% Insert text in the middle of the arrows
node[midway,fill= white] {\large Low constitutive defense};

% Draw the border by line segments
\draw[-, thick] (23.1,7.7) -- (23.1,11.5) -- (29.8,11.5) -- (29.8,7.7) -- (26.8,7.7)-- (26.5,7)
```

```
-- (26.2,7.7) --(23.1,7.7);

% Colorful rectangular box with text
\node[inner sep=0pt] (T1) at (26.5,6.5) [process2]{\large High total herbivore pressure};

\node[inner sep=0pt] (S) at (12,3) []{\includegraphics[width=0.4\columnwidth]{specialists.png}};
% specialists.png is a image file, created seperately.

\draw[->, thick] (11.9, 8.2) -- (11.9, 5.5)
node[midway,fill= white] {\large High constitutive defense};
\draw[-, thick] (8.6,1.7) -- (8.6,5.5) -- (15.3,5.5) -- (15.3,1.7) -- (12.3,1.7) -- (12,1)
-- (11.7,1.7) -- (8.6,1.7);
\node[inner sep=0pt] (T2) at (12,0.5) [process2]{\large High total herbivore pressure};

\node[inner sep=0pt] (O) at (26.5,3) []{\includegraphics[width=0.4\columnwidth]{opt-gen-spec.png}};
% opt-gen-spec.png is a image file, created seperately.

\draw[->, thick] (15.3,8.2) -- (23.1,3.6)
node[midway,fill= white] {\large Optimal constitutive defense};
\draw[-, thick] (23.1,1.7) -- (23.1,5.5) -- (29.8,5.5) -- (29.8,1.7) -- (26.8,1.7) -- (26.5,1)
--(26.2,1.7) -- (23.1,1.7);
\node[inner sep=0pt] (T3) at (26.5,0.5) [process1]{\large Low total herbivore pressure};

% End the code with \end{tikzpicture}.
%%%%%%%%%%%%%%%%%%%%%%%%%%%%%%%%%%%%%%%%%%%%%%%%%%%%%%%%%%%%%%%%%%%%%%%%%%%%%%%%%%%%%%%%%%%%%%%%%

# Instruction: Compile the given latex code in Overleaf.
```

2 Python code of Figure 2

```
#Import packages
import numpy as np
from scipy.integrate import odeint
import matplotlib.pyplot as plt
##################################################################################################

# Define the ODEs and write "t" instead of "D" as independent variable, because odeint by default
take "t" #
def deriv(y, t, alpha, beta, gamma, eta, mu):
    S, G, N = y
    dSdt   =  alpha * t - mu * S * N                        # Attracted specialist population S
    dGdt   =  - beta * G * t - eta * G * N                  # Deterred generalist population G
    dNdt   =  gamma                                         # Atrracted natural enemies N

    return dSdt, dGdt, dNdt

# Insert the parameter values
alpha = 0.04
beta = 0.05
gamma = 0.1
```

```
mu = 0.02
eta = 0.03

# Insert the initial conditions
S0, G0, E0 = 0.0, 20.0, 0.0
t = np.linspace(0, 100, 1500)                                       # Grid of D
y0 = S0, G0, E0                                                     # Initial conditions vector

# Integrate the equations over the time grid, t.
ret = odeint(deriv, y0, t, args=(alpha, beta, gamma, eta, mu))
S, G, E = ret.T

# Define the plots of S, G and N
def plotdc(t, S, G, E):
  f, ax = plt.subplots(1,1,figsize=(8,2), dpi = 300)
  ax.plot (t, S, ’darkcyan’, alpha=0.9, linewidth=3.5, label=’Specialist ($S$)’)
  ax.plot (t, G, ’tomato’, alpha=0.9, linewidth=3.5, label=’Generalist ($G$)’)
  ax.plot (t, E, ’orange’, alpha=0.9, linewidth=3.5, label=’Natural enemies ($N$)’)

# Assign values on x and y axes
  plt.tick_params(axis=’y’, labelsize=13)
  plt.tick_params(axis=’x’, labelsize=13)

# Label on x and y axes
  plt.xlabel(’Plant defense ($D$)’, {"fontsize": 13})
  plt.ylabel(’Populations’, {"fontsize": 13})

# Label the variables (S, G and N) on the top of the Figure
  legend = ax.legend()
  legend.get_frame().set_alpha(0.5)
  plt.legend( bbox_to_anchor=(0., 1.02, 1., .102), loc=’lower left’,
           ncol=2, mode="expand", borderaxespad=0., fontsize=13 )

#Boundary
  for spine in (’top’, ’right’, ’bottom’, ’left’):
      ax.spines[spine].set_visible(True)

      plt.show();

plotdc(t, S, G, E)                                                    # Final figure visualization
##########################################################################################
```

Instruction: Save the code as "Filename.py" and run the python code by the command "python3 Filename.py" in your Linux (Ubuntu) terminal.

3 Python code of Figure 3

```
# Import packages
import numpy as np
import matplotlib.pyplot as plt
```

```
import math
####################################################################################################

# Evenly sampled D at intervals
t = np.arange(0.0, 100.0, 0.02)

# Insert the parameter values
theta = 0.002
delta =0.003
beta = 0.05
alpha = 0.04
G_0 = 20

# Define the plot of Total herbivores (T_H)
f, ax = plt.subplots(1,1,figsize=(8,2), dpi = 300)
ax.plot(t, alpha/theta * (1 - np.exp(- theta * t * t)) + G_0 * np.exp(- (beta + delta) * t * t),
'steelblue', alpha=0.9, linewidth=3.5, label='$T_H$')

# Assign values on x and y axes
plt.tick_params(axis='y', labelsize=13)
plt.tick_params(axis='x', labelsize=13)

# Label on x and y axes
plt.xlabel('Plant defense ($D$)', {"fontsize": 13})
plt.ylabel('$T_H$', {"fontsize": 13})

# Label T_H inside the Figure
legend = ax.legend()
legend.get_frame().set_alpha(0.5)
plt.legend( bbox_to_anchor=(0.78, 0.05, 0.18, 0.1), loc='lower left',
           ncol=1, mode="expand", borderaxespad=0, fontsize=13 )

#Boundary
for spine in ('top', 'right', 'bottom', 'left'):
  ax.spines[spine].set_visible(True)

plt.show();                                                      # Final figure visualization
##################################################################################################

Instruction: Save the code as "Filename.py" and run the python code by the command
"python3 Filename.py" in your Linux (Ubuntu) terminal.
```

4 Python code of Figure 4

```
# Import packages
import numpy as np
import matplotlib.pyplot as plt
import math
##################################################################################################
```

```
# Evenly sampled D at intervals
t = np.arange(0.0, 100.0, 0.02)

# Insert the parameter values
alpha = 0.05
theta = 0.0025
delta =0.001
beta = 0.001
G_0 = 20

# Define the plot of Total herbivores (T_H)
f, ax = plt.subplots(1,1,figsize=(8,2), dpi = 300)
ax.plot(t, alpha/theta * (1 - np.exp(- theta * t * t)) + G_0 * np.exp(- (beta + delta) * t * t),
'indianred', alpha=0.9, linewidth=3.5, label='$T_H$')

# Assign values on x and y axes
plt.tick_params(axis='y', labelsize=13)
plt.tick_params(axis='x', labelsize=13)

# Label on x and y axes
plt.xlabel('Plant defence ($D$)', {"fontsize": 13})
plt.ylabel('$T_H$', {"fontsize": 13})

# Label T_H inside the Figure
legend = ax.legend()
legend.get_frame().set_alpha(0.5)
plt.legend(fontsize=13)

#Boundary
for spine in ('top', 'right', 'bottom', 'left'):
  ax.spines[spine].set_visible(True)

plt.show();                                                        # Final figure visualization
##################################################################################################
```

Instruction: Save the code as "Filename.py" and run the python code by the command "python3 Filename.py" in your Linux (Ubuntu) terminal.

5 Python code of Figure 5

```
# Import packages
import numpy as np
import matplotlib.pyplot as plt
import math
##################################################################################################

# Evenly sampled D at intervals
t = np.arange(0.0, 100.0, 0.02)

# Insert the parameter values
```

```
theta = 0.002
delta =0.003
beta = 0.05

# Define the plot of Total herbivores (T_H)
f, ax = plt.subplots(1,1,figsize=(8,2), dpi = 300)
ax.plot(t, 20 * (1 - np.exp(- theta * t * t)) + 30 * np.exp(- (beta + delta) * t * t), ’olive’,
alpha=0.9, linewidth=3.5, label=’$\u03B1/\u03B8 < G_0$’)          # alpha/theta = 20, G_0 = 30
ax.plot(t, 30 * (1 - np.exp(- theta * t * t)) + 20 * np.exp(- (beta + delta) * t * t), ’peru’,
alpha=0.9, linewidth=3.5, label=’$\u03B1/\u03B8 > G_0$’)          # alpha/theta = 30, G_0 = 20

# Assign values on x and y axes
plt.tick_params(axis=’y’, labelsize=13)
plt.tick_params(axis=’x’, labelsize=13)

# Label on x and y axes
plt.xlabel(’Plant defense ($D$)’, {"fontsize": 13})
plt.ylabel(’$T_H$’, {"fontsize": 13})

# Label T_H inside the Figure
legend = ax.legend()
legend.get_frame().set_alpha(0.5)
plt.legend( bbox_to_anchor=(0.66, 0.05, 0.3, 0.1), loc=’lower left’,
           ncol=1, mode="expand", borderaxespad=0, fontsize=13 )

#Boundary
for spine in (’top’, ’right’, ’bottom’, ’left’):
  ax.spines[spine].set_visible(True)

plt.show();                                                        # Final figure visualization
###################################################################################################
```

Instruction: Save the code as "Filename.py" and run the python code by the command "python3 Filename.py" in your Linux (Ubuntu) terminal.

6 Python code of Figure 6

```
# Import packages
import numpy as np
import matplotlib.pyplot as plt
import math
###################################################################################################

# Evenly sampled D at intervals
t = np.arange(0.0, 100.0, 0.02)

# Insert the parameter values
alpha = 0.04
beta = 0.05
# Different values of theta
```

```
theta1 = 0.002
theta2 = 0.003
theta3 = 0.01
theta4 = 0.008
# Different values of delta
delta1 = 0.003
delta2 = 0.002
delta3 = 0.005
delta4 = 0.001

# Define the plot of Total herbivores (T_H) for different values of theta and delta
f, ax = plt.subplots(1,1,figsize=(8,2), dpi = 300)
# G_0 = 20
ax.plot(t, alpha/theta1 * (1 - np.exp(- theta1 * t * t)) + 20 * np.exp(- (beta + delta1) * t * t),
’y’, alpha=0.9, linewidth=3.5, label=’$G_0 = 20, \u03B8 = 0.002, \u03B4 = 0.003$’)
# G_0 = 30
ax.plot(t, alpha/theta2 * (1 - np.exp(- theta2 * t * t)) + 30 * np.exp(- (beta + delta2) * t * t),
’b’, alpha=0.6, linewidth=3.5, label=’$G_0 = 30, \u03B8 = 0.003, \u03B4 = 0.002$’)
# G_0 = 10
ax.plot(t, alpha/theta3 * (1 - np.exp(- theta3 * t * t)) + 10 * np.exp(- (beta + delta3) * t * t),
’m’, alpha=0.7, linewidth=3.5, label=’$G_0 = 10, \u03B8 = 0.01, \u03B4 = 0.005$’)
# G_0 = 25
ax.plot(t, alpha/theta4 * (1 - np.exp(- theta4 * t * t)) + 25 * np.exp(- (beta + delta4) * t * t),
’g’, alpha=0.7, linewidth=3.5, label=’$G_0 = 25, \u03B8 = 0.008, \u03B4 = 0.001$’)

# Assign values on x and y axes
plt.tick_params(axis=’y’, labelsize=13)
plt.tick_params(axis=’x’, labelsize=13)

# Label on x and y axes
plt.xlabel(’Plant defence ($D$)’, {"fontsize": 13})
plt.ylabel(’$T_H$’, {"fontsize": 13})

# Set legend on the top of the Figure
legend = ax.legend()
legend.get_frame().set_alpha(0.5)
plt.legend( bbox_to_anchor=(0.0, 1.02, 0.63, 0.1), loc=’lower left’,
           ncol=1, mode="expand", borderaxespad=0., fontsize=12 )

#Boundary
for spine in (’top’, ’right’, ’bottom’, ’left’):
   ax.spines[spine].set_visible(True)

plt.show();                                                      # Final figure visualization
####################################################################################################
```

Instruction: Save the code as "Filename.py" and run the python code by the command "python3 Filename.py" in your Linux (Ubuntu) terminal.
